# Parathyroid Hormone-Like Hormone is a Poor Prognosis Marker of Head and Neck Cancer and Promotes Cell Growth via RUNX2 Regulation

**DOI:** 10.1038/srep41131

**Published:** 2017-01-25

**Authors:** Wei-Min Chang, Yuan-Feng Lin, Chia-Yi Su, Hsuan-Yu Peng, Yu-Chan Chang, Jenn-Ren Hsiao, Chi-Long Chen, Jang-Yang Chang, Yi-Shing Shieh, Michael Hsiao, Shine-Gwo Shiah

**Affiliations:** 1 Graduate Institute of Medical Sciences, National Defense Medical Center, Taipei, Taiwan; 2 National Institute of Cancer Research, National Health Research Institutes, Miaoli, Taiwan; 3 Genomics Research Center, Academia Sinica, Taipei, Taiwan; 4 Graduate Institute of Clinical Medicine, College of Medicine, Taipei Medical University, Taipei, Taiwan; 5 Department of Otolaryngology, Head and Neck Collaborative Oncology Group, National Cheng-Kung University, Tainan, Taiwan; 6 Department of Pathology, College of Medicine, Taipei Medical University and Department of Pathology, Taipei Medical University Hospital, Taipei Medical University, Taipei, Taiwan; 7 Department of Internal Medicine, National Cheng-Kung University Hospital, College of Medicine, National Cheng-Kung University, Tainan, Taiwan; 8 Department of Dentistry, Tri-Service General Hospital, National Defense Medical Center, Taipei, Taiwan

## Abstract

Parathyroid Hormone-Like Hormone (PTHLH) is an autocrine/paracrine ligand that is up-regulated in head and neck squamous cell carcinoma (HNSCC). However, the cellular function and regulatory mechanism in HNSCC remains obscure. We investigated the clinical significance of PTHLH in HNSCC patients, and verified the role of RUNX2/PTHLH axis, which is stimulated HNSCC cell growth. In patients, PTHLH is a poor prognosis marker. PTHLH expression lead to increasing the cell proliferation potential through an autocrine/paracrine role and elevating blood calcium level in Nod-SCID mice. In public HNSCC microarray cohorts, PTHLH is found to be co-expressed with RUNX2. Physiologically, PTHLH is regulated by RUNX2 and also acting as key calcium regulator. However, elevations of calcium concentration also increased the RUNX2 expression. PTHLH, calcium, and RUNX2 form a positive feedback loop in HNSCC. Furthermore, ectopic RUNX2 expression also increased PTHLH expression and promoted proliferation potential through PTHLH expression. Using cDNA microarray analysis, we found PTHLH also stimulated expression of cell cycle regulators, namely CCNA2, CCNE2, and CDC25A in HNSCC cells, and these genes are also up-regulated in HNSCC patients. In summary, our results reveal that PTHLH expression is a poor prognosis marker in HNSCC patients, and RUNX2-PTHLH axis contributes to HNSCC tumor growth.

The most fundamental trait of cancer cells is the uncontrolling cell growth^1^. Cancer cells harbor numerous genetic changes to maintain proliferation abilities and resist cell death signals or growth suppressors. Head and neck squamous cell carcinoma (HNSCC) ranks among the eighth of the leading cancers in the USA and is estimated to have reached more than 48,000 new cases in 2016^2^. In the world, there are nearly 300,400 new onset cases and half of the patients will not survive for longer than 5 years^3^. Smoking, alcohol consumption, and HPV infection are the major risk factors for HNSCC^4^. Betel quid is another risk factor of HNSCC in Taiwan, India, and other neighboring countries^5^. Despite the recent advances in cancer treatment that have improved the life quality and expectancy of HNSCC patients, however, the overall survival of HNSCC patients has only improved marginally over the past 30 years^6^. It is therefore important to understand the molecular mechanisms of HNSCC development and progression.

Parathyroid hormone-like hormone (PTHLH) is an autocrine/paracrine ligand that regulates cell differentiation and proliferation, and is expressed in many tissues during development, such as tooth, bone, and mammary gland maturation^7^,^8^. PTHLH interacts with parathyroid hormone/parathyroid hormone-related protein receptor (PTH/PTHLH type 1 receptor, PTH1R), a member of family B G-protein coupled receptor, and controls several cellular function through activation of the cAMP/PKA or IP_3_/PKC signaling cascades. PTHLH promotes cell proliferation, migration, and invasion, and prevents apoptosis in different types of cancers^9^,^10^,^11^. In HNSCC, PTHLH has been reported as a poor prognostic marker and is stimulated *in vitro* cell growth through promoting cell cycle progression^12^. However, the regulation mechanism and tumor progression role of PTHLH in HNSCC remains uncertain.

Runt-related transcription factor 2 (RUNX2) is a major transcription factor that regulates osteoblast differentiation, chondrocyte proliferation, and differentiation in endochondral bone formation process^13^,^14^ and is an important transcription factor in breast and prostate cancer development and progression^15^. In breast cancer cells and normal chondrocytes, RUNX2 stimulates PTHLH expression through Indian Hedgehog (IHH) expression or direct binding to PTHLH promoter with GLI2 complex^16^,^17^,^18^. Silencing RUNX2 also inhibits PTHLH expression in breast cancer cells^19^. However, the role of RUNX2-PTHLH axis has not been studied in HNSCC.

In this study, we showed that PTHLH promoted HNSCC growth through an autocrine/paracrine manner and could serve as a poor prognosis marker in HNSCC patients. Secondly, concordant up-regulation of PTHLH and RUNX2 promoted HNSCC tumor growth, and RUNX2 was stimulated by calcium level. Finally, PTHLH elevated the blood calcium level in Nod-SCID mice and stimulated expression of several cell cycle regulators, namely, CCNA2, CCNE2 and CDC25A, in HNSCC cells and patients. In summary, our study not only reveals a novel positive feedback loop among RUNX2, PTHLH, and calcium but also shows RUNX2/PTHLH axis promotes HNSCC tumor growth.

## Results

### PTHLH overexpression is correlated with poor prognosis in HNSCC patients

**Figure 1:**
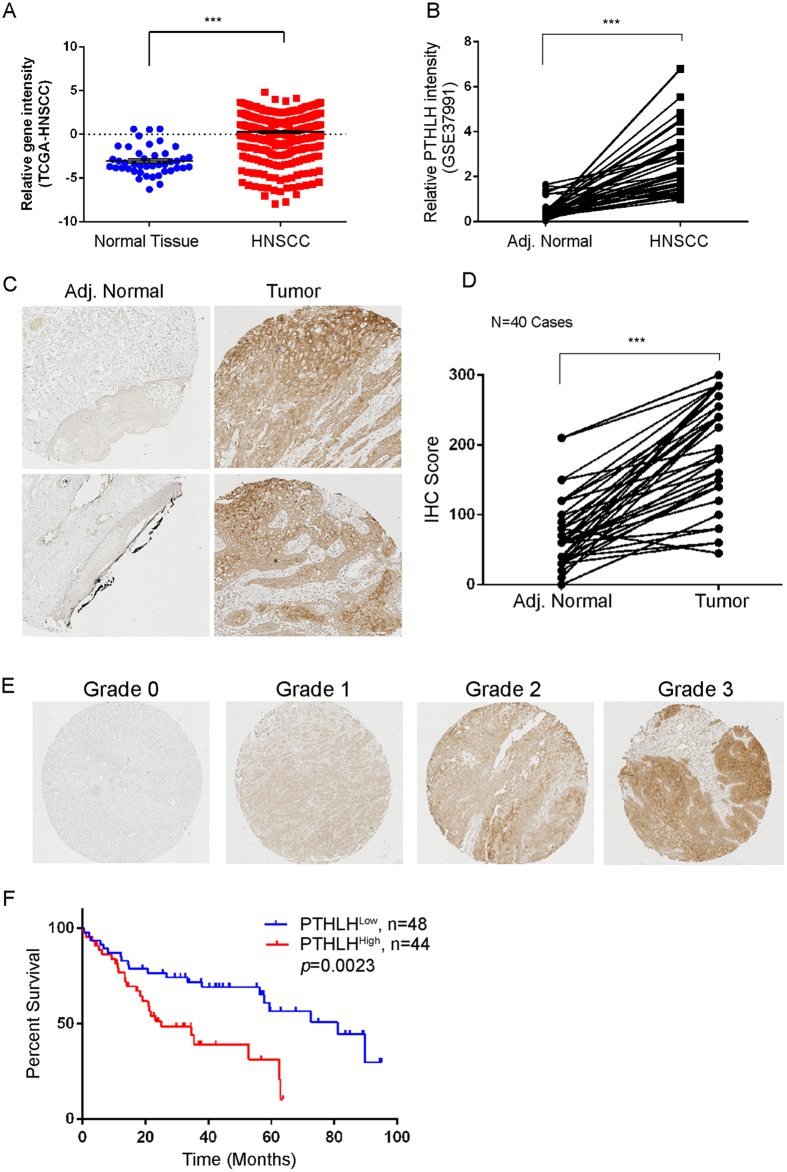
PTHLH is a poor prognostic marker in HNSCC. (**A** and **B**) Clinical RNA expression profile of PTHLH in TCGA (**A**) and Taiwanese HNSCC cohort (**B**, GSE37991). (**C**) Representative images from IHC staining of PTHLH protein levels in matched primary head and neck tumors and adjacent normal tissues. (**D**) Quantification of cytoplasmic IHC expression of PTHLH in primary head and neck tumors in comparison with paired adjacent normal tissues. The scores are calculated as staining intensity multiplied by percentage of stained cells. ***p < 0.001. (**E**) Scores indicating PTHLH levels in representative head and neck tumor tissues. (**F**) Kaplan–Meier plots of overall survival of 92 patients. The differences between groups were tested using log rank tests.

To elucidate the clinical relevance of PTHLH in HNSCC patients, we first analyzed PTHLH mRNA expression profiling from the TCGA Data Portal. The PTHLH expression levels are significantly higher in primary tumors compared with normal solid tissue (Fig. 1A, *p* < 0.001). We also examined the PTHLH expression in a paired Taiwanese HNSCC microarray cohort (GSE37991)^20^ and the Oncomine™ database. PTHLH expression was up-regulated in HNSCC tumors comparing to normal control tissues (Fig. 1B and Supplementary Figure s1). Intriguingly, PTHLH is the second and fifteenth highest up-regulated gene in HNSCC tumors (Supplementary Figure s1A and B). This phenomenon indicates that PTHLH is predominantly expressed in HNSCC patients and might be easily detected from patients. We further analyzed PTHLH protein level in an independent testing set HNSCC tissue array cohort with 92 specimens by using immunohistochemistry (IHC) staining. We compared 40 paired samples from primary head and neck tumors and normal adjacent tissues in this tissue array. Strikingly, in 35 of 40 patients (~88%), the PTHLH protein level was significantly higher in tumors than adjacent normal tissues (Fig. 1D, *p* < 0.001). Next, we tested whether PTHLH expression could be a poor prognosis marker in HNSCC patients. Typical PTHLH staining patterns in HNSCC tumors of the defined scoring criteria are shown in Fig. 1E. Our results reveal that patients with high PTHLH expression (score 2 or 3) have a significantly shorter survival time than those with low PTHLH expression (score 0 or 1, *p* = 0.0023), and the mean survival time is 25.1 months and 81.2 months, respectively. Taken together, these results demonstrate that PTHLH is up-regulated and could be a poor prognostic marker in HNSCC patients.

### Autocrine/paracrine PTHLH promotes HNSCC growth abilities

**Figure 2:**
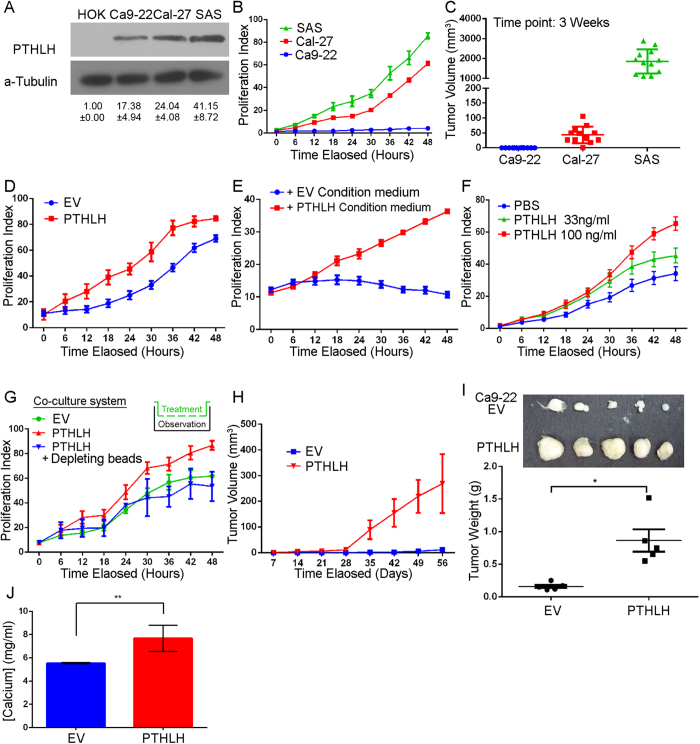
Autocrine PTHLH controls HNSCC cell growth. (**A**) PTHLH expression among primary oral keratinocyte (HOK) and HNSCC cell lines was analyzed by Western blotting. PTHLH bands were cropped from Supplementary Figure s5A at 17 kDa region and internal control, a-Tubulin were cropped from 55 kDa marker. (**B** and **C**) The *in vitro* proliferation (**B**) and *in vivo* tumor growth (**C**) abilities in HNSCC cells. (**D**) Cellular proliferation abilities of Ca9-22 cells without (empty vector, EV) or with PTHLH overexpression. (**E** and **F**) Cellular proliferation abilities of Ca9-22 cells treatment with condition medium of parental Ca9-22 or PTHLH overexpression cells (**E**) and recombinant PTHLH protein (**F**). (**G**) Cellular proliferation abilities of Ca9-22 cells that were co-cultured with c-terminal histag-PTHLH overexpression cells or were combined with nickel depleting beads. (**H**) The *in vivo* tumor growth abilities of Ca9-22 cells without (EV) or with PTHLH overexpression in Nod-SCID mice (n = 5). (**I**) Upper figure is the paired tumor image and lower panel is the tumor weight of (**H**). (**J**) The blood calcium level of (**H**). *p < 0.05, **p < 0.001. In (**B**,**D**,**E**,**F**,**G** and **J**) data from three independent experiments were presented as mean ± SEM. The statistical significance was analyzed by ANOVA test.

Our clinical findings suggest that PTHLH may play an important role in HNSCC progression. In order to investigate the PTHLH role in HNSCC progression, we compared the endogenous PTHLH level among the primary oral keratinocyte (HOK) and HNSCC cells, namely, Ca9-22, Cal-27 and SAS. All HNSCC cells have higher endogenous PTHLH protein level than HOK (Fig. 2A). Furthermore, we examined *in vitro* proliferation and *in vivo* tumor growth abilities among those HNSCC cells. Cal-27 and SAS cells that have higher endogenous PTHLH protein levels than Ca9-22 cells (Fig. 2A) also harbor higher growth potential than Ca9-22 cells. Both Cal-27 and SAS cells have higher *in vitro* proliferation abilities (Fig. 2B, *p* < 0.001) and larger tumor burden after a 3 week inoculation in SCID-mice (Fig. 2C, SAS and Cal-27 verse Ca9-22, *p* < 0.001). These results indicate that PTHLH might correlate with HNSCC growth. We then enforced PTHLH expression in Ca9-22 cells to examine if PTHLH would promote HNSCC tumor growth. Indeed, the Ca9-22-PTHLH cells have higher *in vitro* proliferation abilities (Fig. 2D, *p* < 0.001) and *in vivo* tumor growth rate (Fig. 2H, *p* < 0.001) than empty vector (EV) cells. Additionally, PTHLH has been reported to elevate serum calcium level^21^. Therefore, we measured mice serum calcium level in Ca9-22 inoculation mice. Ca9-22-PTHLH cells also have elevated blood calcium level than Ca9-22-EV cells (Fig. 2J, *p* < 0.001). To further investigate the role of PTHLH in HNSCC growth, we examined whether PTHLH would promote HNSCC cell growth through an autocrine/paracrine manner. We used the condition medium harvested from Ca9-22-PTHLH or Ca9-22-EV cells. Only the Ca9-22-PTHLH condition medium stimulates the parental Ca9-22 growth (Fig. 2E, *p* < 0.001). To observe the autocrine/paracrine PTHLH function in HNSCC cell growth, we used a transwell co-culture system, which can mimic the autocrine or paracrine role of PTHLH and the co-culture system can be easily depleted PTHLH from co-culture system. PTHLH-expressed cells stimulated the parental Ca9-22 growth comparing to empty vector, and this phenomenon was abolished by PTHLH depletion (Fig. 2F, *p* < 0.05). Finally, we treated the parental Ca9-22 cells with recombinant PTHLH protein. PTHLH also stimulated the parental Ca9-22 growth in a dose-dependent manner (Fig. 2G, *p* < 0.05). These results indicate that PTHLH stimulates HNSCC cell growth through an autocrine/paracrine manner and PTHLH-expressed HNSCC cells also have an elevated blood calcium level in Nod-SCID mice.

### RUNX2 expression is positively correlated with PTHLH expression in HNSCC patients

**Figure 3:**
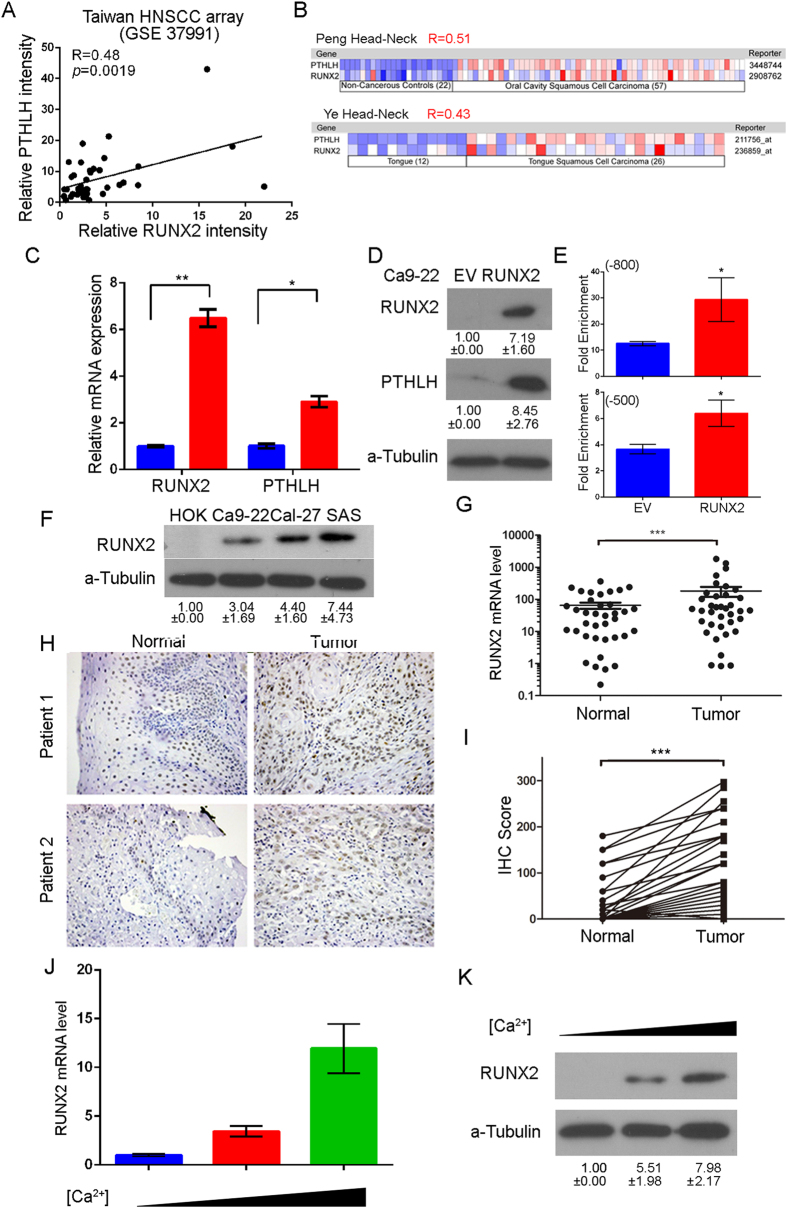
RUNX2 regulates PTHLH expression and RUNX2 is overexpressed in HNSCC tumor. (**A**) The mRNA expression correlation between RUNX2 and PTHLH in Taiwanese HNSCC microarray database (GSE37991) (**B**) The mRNA expression correlation between RUNX2 and PTHLH in HNSCC from the Oncomine™ database. The Pearson’s Correlation test was used to evaluate the statistical significance of correlation between RUNX2 and PTHLH expression. (**C** and **D**) PTHLH mRNA (**C**), cellular protein (**D**) expression after the enforced expression of ectopic RUNX2 in Ca9-22 cells. The RUNX2 band was cropped from Supplementary Figure 5B at 55 kDa region. (**E**) RUNX2 ChIP-qPCR results on −800 bp and −500 bp RUNX2 binding regions of PTHLH promoter in Ca9-22 cells after enforced expression of ectopic RUNX2. (**F**) RUNX2 expression among HOK and HNSCC cell lines was analyzed by Western blotting (Supplementary Figure 5C). (**G**) RUNX2 mRNA levels in in 45 paired adjacent normal tissues and tumor tissues from HNSCC patients. RUNX2 protein levels in 40 paired adjacent normal tissues and tumor tissues from HNSCC patients. (**H**) Representative images from IHC staining of RUNX2 from paired HNSCC tissues. (**I**) The quantification of IHC results of RUNX2 IHC. (**J** and **K**) The RUNX2 mRNA (**J**) and protein (**K**, Supplementary Figure 5D) expression after exposure to different concentration calcium cation (1.8, 2.4, and 3.0 mM). In (**C**,**E**,**G**,**I** and **J**) data from three independent experiments were presented as mean ± SEM. The statistical significance was analyzed by Student t-test. (*p < 0.05; **p < 0.01; ***p < 0.001).

Recently, RUNX2 has been reported to regulate PTHLH expression in chondrocyte and breast cancer cells^16^,^17^,^18^. In Taiwanese HNSCC patients, we found that RUNX2 expression is positively correlated with PTHLH level (Fig. 3A, R = 0.48; *p* = 0.0019). Furthermore, we affirmed this positive correlation between RUNX2 and PTHLH in two Oncomine™ HNSCC datasets (Fig. 3B, R = 0.51 and R-0.43), which are the same cohorts that PTHLH belonged to the top 1% up-regulated genes. These results indicate that RUNX2 might regulate PTHLH expression in HNSCC patients. The endogenous RUNX2 level is positively correlated with endogenous PTHLH level in HNSCC cells (Figs 2A and 3F). Next, we enforced the expression of RUNX2 in Ca9-22 cells to show whether the RUNX2 protein could promote PTHLH expression. When RUNX2 was overexpressed, the PTHLH mRNA (Fig. 3C, *p* < 0.001), protein (Fig. 3D, *p* < 0.001), and secretion PTHLH (Supplementary Figure s2, *p* < 0.001) were all up-regulated in Ca9-22-RUNX2 cells comparing to empty vector control cells. In order to confirming ifRUNX2 directly or indirectly regulates PTHLH expression in HNSCC cells, we preformed the chromatin immunoprecipitation (ChIP) followed by q-PCR and immunoprecipitation for Runx2 in Ca9-22 cells. Our results revealed that, in Ca9-22 RUNX2 cells, RUNX2 directly binds to −800 bp and −500 bp region upstream of transcription start site on PTHLH promoter (Fig. 3E, *p* < 0.05) without collaborating with Gli2 (Supplementary Figure s3) even though it is concurrently recruited to regulate Runx2-mediated PTHLH expression in breast cancer^16^.

### RUNX2 is up-regulated in HNSCC patients and stimulated by calcium

In paired HNSCC samples of both real-time PCR and IHC results reveal that RUNX2 mRNA (Fig. 3G, 22/43 = 51.0%, p < 0.001) and protein (Fig. 3H and I, 30/38 = 78.9%, p < 0.001) are significantly up-regulated in clinical HNSCC tumors. Slaked lime (calcium hydroxide) is a common additive in betel nuts, and most of our HNSCC patients are betel nut chewers. During osteoblast differentiation and formation, calcium level also stimulates RUNX2 expression^22^. For the sake of clarity, we further examined whether calcium could stimulate RUNX2 expression in HNSCC cells. We treated Ca9-22 cells with different concentrations of calcium, and both RUNX2 mRNA expression and protein level increases accompany with calcium level (Fig. 3J and K). Taken together, these results demonstrate that up-regulation of RUNX2 in HNSCC patients might be stimulated by calcium from slaked lime.

### RUNX2-PTHLH axis stimulates HNSCC tumor growth

**Figure 4:**
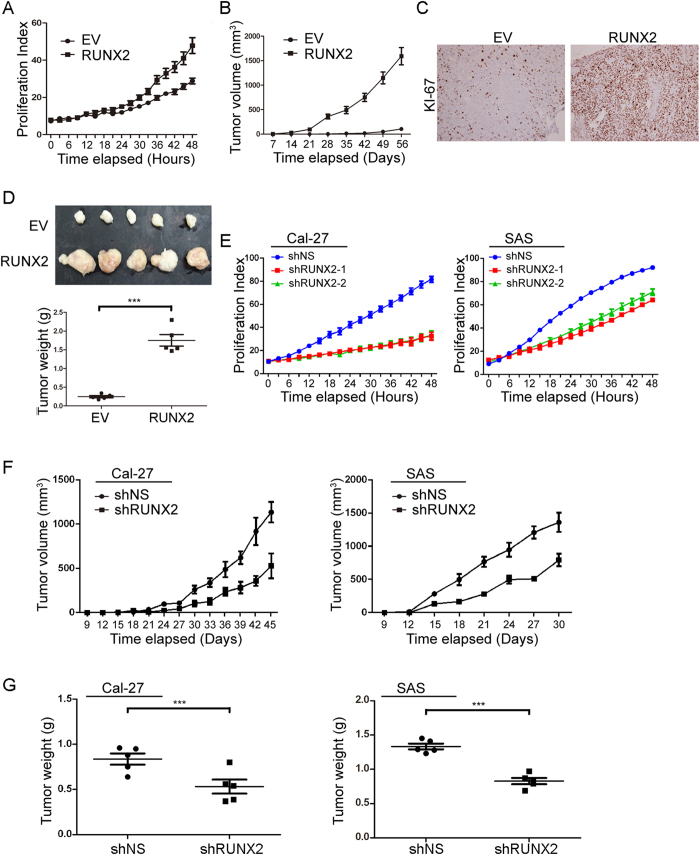
RUNX2 controls HNSCC cell growth. (**A** and **B**) The *in vitro* proliferation (**A**) and *in vivo* tumor growth (**B**) abilities in of Ca9-22 cells without (EV) or with RUNX2 overexpression. (**C** and **D**) The KI-67 staining result (**C**) and tumor image and weight (**D**) of figure (**B**). (**E** and **F**) The *in vitro* proliferation (**E**) and *in vivo* tumor growth (**F**) abilities of Cal-27 and SAS cells stably infected with non-silencing (NS) or 2 independent RUNX2 shRNA clones. (**G**) Tumor weight result of (**F**). In (**A**,**B**,**D**,**E,F** and **G**) data from three independent experiments were presented as mean ± SEM. The statistical significance was analyzed by Student t-test or ANOVA test. ***p < 0.001.

**Figure 5:**
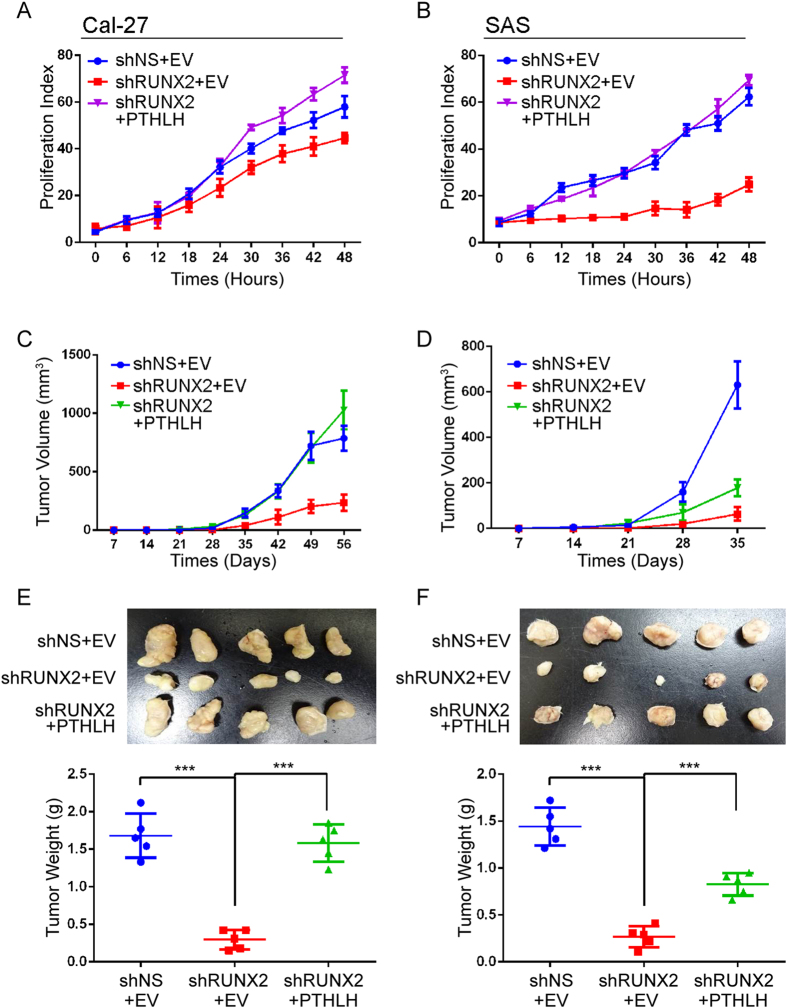
RUNX2 regulates PTHLH to promote HNSCC tumor growth. (**A** and **B**) *In vitro* proliferation assay for the RUNX2-PTHLH axis. The restoration of PTHLH was performed in RUNX2-silencing Cal-27 (**A**) and SAS (**B**) cells. (**C** and **D**) *In vivo* tumor growth assay of Cal-27 (**C**) and SAS (**D**) from (**A** and **B**, n = 5). (**E** and **F**) The tumor images and tumor weights from (**C** and **D**) respectively. The statistical significance was analyzed by Student t-test or ANOVA test. ***p < 0.001.

To examine whether RUNX2 also controls the HNSCC growth and PTHLH is the major contributor in HNSCC proliferation, we enforced the expression of RUNX2 in Ca9-22 cells. The results show RUNX2 promoted *in vitro* proliferation abilities (Fig. 4A, *p* < 0.001) and *in vivo* tumor growth (Fig. 4B, *p* < 0.05 and 4D, *p* < 0.001). Furthermore, the KI-67 proliferation index also reveal that RUNX2 increases *in vivo* proliferation abilities in Ca9-22 cells comparing to control cells (Fig. 4C, *p* < 0.001). Conversely, we also performed complementary studies in Cal-27 and SAS cells. The *in vitro* proliferation abilities of Ca-27 and SAS cells were dramatically decreased in two independent RUNX2 knockdown clones comparing to non-targeting scramble (NS) control cells (Fig. 4E, *p* < 0.01). We further used the shRUNX2-1 clone to measure the *in vivo* tumor growth abilities governed by RUNX2. Both tumor burden (Fig. 4F, *p* < 0.05) and tumor weight (Fig. 4G, *p* < 0.001) were reduced by silencing RUNX2 expression. In addition, roxithromycin, which suppresses RUNX2 mRNA expression in HNSCC cells^23^, inhibited the Cal-27 and SAS growth (Supplementary Figure s4). To show the RUNX2-PTHLH axis promotes HNSCC cancer growth, we restored PTHLH expression in RUNX2-silent Cal-27 and SAS cells. PTHLH was predominantly restored the *in vitro* proliferation abilities (Fig. 5A and B, *p* < 0.05) and *in vivo* tumor growth abilities (Fig. 5C–F, *p* < 0.05). Interestingly, PTHLH only fully rescued the tumor growth abilities in Cal-27 cells, but not in SAS cells, after Runx2 knockdown. By using Ingenuity Pathway Analysis software to perform differential display of gene expression obtained from Genomics of Drug Sensitivity in Cancer (GDSC) database between Cal-27 and SAS cells^24^. We found that Ca^2+^ acts as an upstream regulator for Cal-27 cells, but not in SAS cells (Supplementary Table s2), and several down-stream genes of Ca^2+^-related pathway were also up-regulated in Cal-27 cells (Supplementary Table s3). This observation might be able to interpret the different response in those cells. Taken together, these results reveal that RUNX2 governs the proliferation abilities through PTHLH.

### Knowledge-based analysis of the microarray data reveals that PTHLH stimulates cell cycle regulator expression

**Figure 6:**
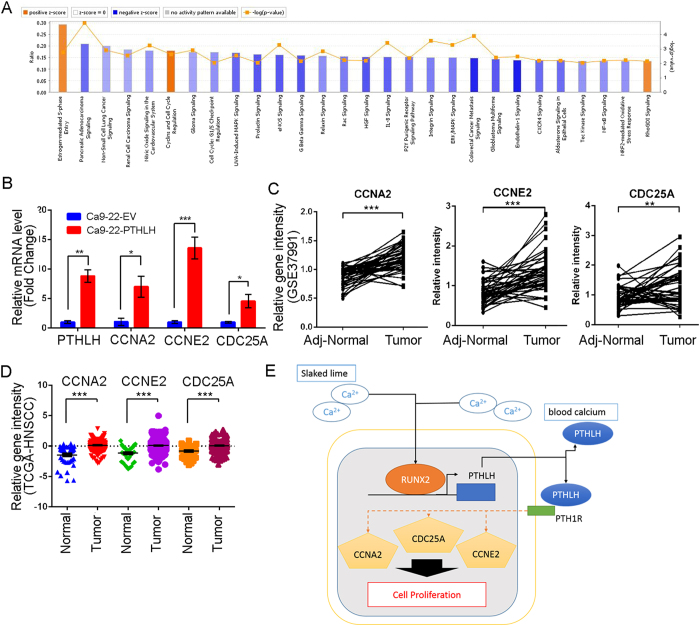
PTHLH stimulates cell cycle regulators, leading to overexpression, in HNSCC cells and patients. (**A**) The most statistically significant pathways associated with PTHLH overexpression in Ca9-22 cells. Orange is activation pathways and blue is suppression. (**B**) mRNA expression after the enforced expression of ectopic PTHLH in Ca9-22 cells. (**C** and **D**) Clinical RNA expression profile of PTHLH stimulation cyclins and cell cycle regulation genes in Taiwanese HNSCC cohort (**C**, GSE37991) and TCGA cohort (**D**). (**E**) Hypothetical model of RUNX2-PTHLH positive loop in HNSCC tumor growth. In (**B**) data from three independent experiments were presented as mean ± SEM. The statistical significance was analyzed by Student t-test. (*p < 0.05; **p < 0.01; ***p < 0.001).

To ascertain the mechanism that PTHLH stimulated HNSCC growth and impacted the clinical HNSCC patients. Therefore, we performed overexpression analysis of PTHLH in Ca9-22 cells with microarray, and then conducted Ingenuity Pathway Analysis (IPA). The PTHLH gene signature is defined as absolute fold change ≥1.5 folds in PTHLH overexpressed cells. The bioinformatics results show that PTHLH overexpression activated the cell cycle regulation pathway (Supplementary Table s1), such as “Cyclins and Cell Cycle Regulation” (z score = 1.941 and –log*P* value = 2.640) and “Estrogen-mediated S-phase Entry” (z score = 1.633 and −log*P* value = 2.770). These results suggest that PTHLH promotes HNSCC tumor growth through regulation of the cell cycle key molecules expression. Moreover, we examined the expression status of those key cell cycle molecules among clinical HNSCC patients (Supplementary Table s4). We found that CCNA2, CCNE2 and CDC25A are significantly up-regulated in PTHLH expression Ca9-22 cells (Fig. 6B) and those are also up-regulated in the in Taiwanese (Fig. 6C, *p* < 0.01) and TCGA data (Fig. 6D, *p* < 0.001) HNSCC tumor. Therefore data from clinical HNSCC patients also demonstrate that PTHLH might regulate tumor growth through cell cycle regulators, namely, CCNA2, CCNE2, and CDC25A (Fig. 6E). In summary, PTHLH is a key stimulator that promotes several key cell cycle regulator expression in HNSCC. This phenomenon is consistent with our observation of PTHLH function in HNSCC growth.

## Discussion

Aberrant cell proliferation is the foundation of cancer growth and progression; therefore, realization of novel growth-stimulating molecules is crucial. Secretion of PTHLH has been reported to play a vital role in tumorigenesis, cancer progression, hypercalcemia, and also controlling several tumor relevant genes expression^25^,^26^,^27^. Both RUNX2 and PTHLH may play certain roles in tumor cell transformation, growth, metastasis, hypercalcemia, and cachexia^28^,^29^. PTHLH has been reported as a poor prognosis marker of HNSCC^12^,^30^, however, the role of PTHLH in HNSCC progression is unclear. In this study, we investigated the role of PTHLH in head and neck cancer by analyzing its expression in clinical patients and its phenotypic impact by investigating *in vitro* and *in vivo* cell growth due to PTHLH in HNSCC. Previously we showed that over-expression of RUNX2 in Ca9-22 cells promotes the PTHLH mRNA level in microarray studies^31^ and the positive correlation of endogenous PTHLH and RUNX2 protein level (Figs 2A and 3F) causally affects the cancer progression in HNSCC cells. Whereas Ca9-22 cells with low PTHLH level displayed a poorer *in vitro* and *in vivo* tumor growth (Fig. 2B and C) and metastatic potentials^31^, Cal-27 and SAS cells that express higher PTHLH levels exhibited a more aggressive capacity in tumorigenesis and metastasis. Thus, we used those HNSCC cell lines to perform the *in vitro* and *in vivo* models for clarifying the role of RUNX2 in regulating the transcription of PTHLH during HNSCC progression in this study. Furthermore, we found high PTHLH expression was associated with short survival time in HNSCC patients, and PTHLH promoted HNSCC growth and blood calcium level. Using ChIP-qPCR assay, we demonstrated that RUNX2 binds directly to PTHLH promoter and thereby stimulates PTHLH expression (Fig. 3E) which appears to be important for promoting tumor growth in Ca9-22 cells.

PTHLH activates the G-protein coupled receptor, PTH1R, which subsequently activates cyclic AMP induced protein kinase A (PKA) signaling and cytosolic calcium induced protein kinase C (PKC) pathway^7^. In many cancer types, such as breast, prostate, and renal cell carcinoma, PTHLH is highly expressed and plays a role in bone metastasis and osteolysis^32^,^33^,^34^. In HNSCC, PTHLH is reported to be up-regulated and contributes to cancer malignancy^35^,^36^,^37^. Interestingly, PTHLH also harbors a nuclear localization signal in its peptide sequence, and the wild type but not NLS-mutated PTHLH promotes cell cycle^38^. Based on our IHC staining results, cytosolic PTHLH is the most abundant form while nuclear PTHLH is less abundant, and autocrine/paracrine PTHLH is the dominant form in HNSCC patients. This results provide evidence that autocrine PTHLH might stimulate HNSCC cells growth. Secreted PTHLH is the dominant form in HNSCC, it is therefore easy to detect and could be a therapy niche. Mak *et al*. has used the neutralized PTHLH antibodies to prevent *in vitro* bone tumor proliferation and induced apoptosis^39^. Target to PTHLH may provide a new therapy niche for HNSCC patients.

In addition, we found PTHLH is important in regulating cell cycle progression. Cyclins such as CCNA2 and CCNE2 and M-phase inducer phosphatase 1 (CDC25A) are key genes that are stimulated by PTHLH and they contribute to bypass cell cycle check point and phase transition. CCNA2 serves as a poor prognosis marker to reduce overall survival time and disease-free survival time in HNSCC patients who have undergone surgery and postoperative radiotherapy^40^. CCNE2 is the rate limiting molecules in G1 to S transition, and dysregulation of CCNE2 has a potent role in tumorigenesis^41^. CDC25A is one of the most crucial cell cycle regulators in controlling G1/S and G2/M entry that enhance mitosis and tumor growth^42^. All these genes are key cell cycle regulators that are also up-regulated in clinical HNSCC patients. Taken together, PTHLH is the up-stream activator that governs the cell cycle progression. Therefore, therapy agents such as roxithromycin, which has inhibited RUNX2 and PTHLH expression, might prevent the HNSCC proliferation and other PTHLH induced cancer related complications.

The additives of betel nut, slaked lime, elevate the local calcium concentration and damage buccal mucosa. Furthermore, RUNX2 level is elevated under hyper calcium condition^43^, and PTHLH is regulated by CaR signaling^44^. PTHLH mediates multiple effects on osteoblast and osteoclast function and is responsive to circulating calcium concentration^45^. In cancer patient, aberrant PTHLH expression is the predominant cause of hypercalcemia as well as cancer cachexia. Over 80 percent of the cancer patients with hypercalcemia have an increased serum PTHLH concentration^46^. This result is consistent with our observation that PTHLH increases the mice blood calcium concentration. Taken together, calcium, RUNX2, and PTHLH form a positive feedback loop that may increase protein expression of RUNX2/PTHLH axis and promote the *in orthotopic* tumor growth by an autocrine/paracrine PTHLH manner. Moreover, PTHLH may promote cancer hypercalcemia and cachexia in HNSCC patients. Thus, reducing the PTHLH expression or local calcium supplement from slaked lime might prevent disease progression.

In conclusion, we demonstrated that PTHLH is overexpressed in HNSCC comparing to adjacent normal tissues, and is a poor prognosis marker of HNSCC. PTHLH expression is controlled by RUNX2 and is correlated with head and neck cancer growth. In HNSCC patients, PTHLH expression might stimulate cell cycle pathways and positively regulate key protein expression. We speculate that PTHLH could be a biomarker for HNSCC progression and may be a potential therapeutic target for head and neck cancer patients.

## Methods

### Patient and ethics statement

Paired RNAs from HNSCC tumor specimens and adjacent non-cancerous epithelia were obtained from surgeries performed between 1999 and 2010 at the National Cheng Kung University Hospital. Frozen tissues were preserved in liquid nitrogen. The American Joint Committee on Cancer (AJCC) staging system was used for tumor staging^47^. The study protocol was approved by the Institutional Human Experiment and Ethics Committee of National Health Research Institutes (HR-97-100). The independent validation HNSCC cohort in formalin-fixed, paraffin-embedded HNSCC tissue microarray data was collected from Taipei Medical University Hospital with IRB approval (TMU-IRB 99049) for further immunohistochemistry (IHC) analysis for cancer biomarker. Archived specimens were spotted onto tissue microarrays on Dako coated slides before being used for IHC staining. The histological diagnosis of oral cancer was performed according to the WHO classification and recommendations. Primary tumor size, local invasion, distal metastasis, lymph node involvement, and the final disease stage were determined according to the definition of the AJCC TNM staging system of oral cancer^47^. Conditions of the patients were followed for up to 100 months.

All study was carried out in accordance with the approved guidelines. No informed consent was required because the data were analyzed anonymously and no identifying information relating to participants were included.

### Immunohistochemical staining and interpretation

PTHLH, RUNX2 and Ki-67 IHC staining was performed using an automated immunostainer (Ventana Discovery XT autostainer, Ventana, USA). Antigens were retrieved by heat-induced antigen retrieval for 30 minutes with TRIS-EDTA buffer. Slides were stained with polyclonal rabbit PTHLH antibody (1:200; GeneTex, Taiwan), monoclonal mouse RUNX2 antibody (1:20; Santa Cruz Biotechnology, CA), and monoclonal mouse Ki-67 antibody (1:100; Dako, Denmark).

For PTHLH IHC staining interpretation, both the immunoreactivity intensity and percentage were recorded. The intensity of staining was defined as 0, no staining; 1+, weak staining; 2+, moderate staining; 3+, strong staining. The extent of staining was scored by the percentage of positive cells (0–100%). The final IHC scores (0–300) were the results of staining intensity score multiplied by the percentage of positive cells. Then, all cases were divided into two groups according to the final IHC scores. A score more than and include 150 itself was defined as high IHC expression level and a score less than 150 was defined as low expression.

### Cell culture and materials

HNSCC cells were prepared and maintained according to a standard protocol. The primary human oral keratinocytes (HOKs) were obtained from ScienCell (Carlsbad, CA) and passaged according to the manufacturer’s instructions. 293 T and Cal-27 cells were obtained from American Type Culture Collection. Ca9-22 and SAS cells were obtained from JCRB Cell Bank at 2014 and maintained in accordance with the manufacturer’s instructions. All cells were routinely checked on the morphology and growth characteristics as well as by STR analysis and mycoplasma tests. Roxithromycin (RXM) was purchased from Sigma.

### Lentiviral knockdown and cDNA expression vector and lentivirus package

Lentiviral pGIPZ non-silencing control (NS), shRUNX2 and shPTHLH knockdown clones were purchased from openbiosystem (Thermo-Fisher, Bremen, Germany). RUNX2 and PTHLH donor cDNA vectors were purchased from DNASU^48^ then *in vitro* recombined into pLenti6.2-DEST or pLenti6/capTEV™-CT-DEST vector with gateway LR clonase II kit (Invitrogen-Gibco, NY). All the vectors were confirmed with Sanger sequencing. Lentiviral vectors were transfected into the packaging cell line 293 T with the pCMVΔR8.91 and pMD.G plasmids using a calcium phosphate transfection kit (Invitrogen-Gibco, NY). The viral soups infected to the target cells for 48 hours incubation, then the infected cells were cultured in the optimal concentrations of puromycin or blasticidin (Calbiochem, La Jolla, CA), depending on the vector backbone and cell characteristics.

### cDNA Reverse transcription and real-time PCR gene amplification analysis

RNA of the patients was extracted from formalin-fixed, paraffin-embedded and amplified with cRNA amplification kit. 500 ng cRNA or total RNA from cancer cells then reverse transcribed with superscript III reverse transcriptase (Invitrogen-Gibco, NY). Patient RUNX2 expression was examined by UPL system (Roche, Switzerland) with specific primer (Table s3) and normalized with GAPDH. Other gene expression assays were detected by OmicsGreen (Omics Bio, Taipei, Taiwan).

### Gene expression microarray experiment

Ca9-22 cells were infected with empty vector or PTHLH vector then enriched with blasticidin selection. Total RNA was extracted by QIAGEN RNeasy mini kit. The PTHLH stimulated genes in HNSCC were performed by Affymetrix U133 microarray assays and upload into GEO database (GSE81471).

### Western blot and Enzyme-linked immunosorbent assay of PTHLH expression

Western blot analyses were performed with BioRad mini-protein 3 SDS-PAGE system and data acquired from Fujifilm X-ray films or LAS-3000 Imaging System. PTHLH ELISA was purchased from Cloud-Clone Crop. (Houston, TX). For the ELISA assays, a stable HNSCC cells were seeded into a 6-cm dish. After the cells reach to 80% confluence, the serum-free medium were changed and incubated for 24 hours. Cell debris was removed from the condition medium then stored at −80 °C. The entire condition medium was measured within one month and following the instruction manual.

### Chromatin immunoprecipitation quantitative PCR (ChIP-Q-PCR) and immunoprecipitation assay

ChIP was performed using both the EZ-Zyme chromatin prep kit (Millpore) and ab500 ChIP kt (Abcam) with 10 μg primary RUNX2 antibodies (sc-10758X, Santa cruz) and rabbit IgG controls (Millpore) with 1 mg Ca9-22 EV and Ca9-22-RUNX2 cell lysates. The specific gene and amplicon expression was detected with OmicsGreen. The immunoprecipitation assay was performed by the same condition as stated in ChIP assay. The whole cell lysates derived from Ca9-22 cells were incubated with 10 μg of IgG control or RUNX2 primary antibodies followed by the precipitation with 20 μl of PureProteome™ Protein A/G Mix Magnetic Beads then blot with Gli2 antibodies (1:1000, Cell Signaling). Antibodies and primers were listed in Table s5.

### Incucyte Cell Proliferation assay and transwell co-culture system

For the cell proliferation assays, a stable mixture of OSCC cells (1 × 10^4^ cells) were seeded into a 24-well plate with 0.5 ml of culture medium, and the cells were allowed to attach for at least 2 hours. After the cells had attached, cellular confluence was recorded using the IncuCyte™ Kinetic Live Cell Imaging System (Essen BioScience, Ann Arbor, MI) every 6 hours. PTHLH condition medium was prepared as ELISA samples and recombinant PTHLH was purchased from PeproTech (Rocky Hill, NJ). The transwell co-culture assays were performed with Millicell^®^ cell culture inserts (Merck-Millipore) and depleted the PTHLH with the nickel-NTA agaroses (QIAGEN). Briefly, the 5 × 10^4^ stable cell expressed c-terminal his-tag fusion PTHLH or empty vector were seeded in the upper insert and 1 × 10^4^ parent Ca9-22 cells were seed in the lower wells and assayed cell proliferation by IncuCyte system.

### Animal studies

Non-obese diabetic severe combined immunodeficiency (Nod-SCID) mice are severe immunodeficiency genetic disorder mice and have impaired adaptive immunity development that are excellent recipient mouse model for engraftment human cancer cells^49^. Thus, we decided to use Nod-SCID mice for the *in vivo* animal studies. All animal experiments were performed in strict accordance with the guidelines of the Care and Use of Laboratory Animals. The animal protocol was approved by the Institutional Animal Care and Use Committee of the Genomic Research Center, Academia Sinica (Protocol No: AS-IACUC-15-06-833). Male Nod-SCID mice at the age of 5–6 weeks were bred in the Genomic Research Center. The animals were housed in a climate-controlled room with 12:12 dark-light cycle, and constant temperature and humidity, and food and water provided *ad libitum*. All efforts were made to minimize pain suffering. For the tumor burden assay, 5 × 10^6^ stable OSCC subline cells were resuspended in sterile phosphate-buffered saline (PBS) then injected subcutaneously (SC) into the right flank of the mice. Each group consists of 5 animals. The tumor burden was measured with the following formula: tumor volume (V) = 0.5 × L × W^2^. The mice were sacrificed and the tumors were weighed and photographed. The mice blood calcium levels were measured by Biovision Calcium Colorimetric Assay Kit.

### Statistical analysis

Paired t-tests were performed to compare the RUNX2 and PTHLH IHC expression levels, and RUNX2 mRNA level in cancer tissues and the corresponding adjacent normal tissues. The survival rate of HNSCC patients was calculated by the Kaplan-Meier method and compared by using log-rank test. Patient follow-up times were censored until 100 months. The Western blotting results were quantified by Image J and presented the mean (±SD) from three independent experiments. Bar graphs also present the mean (±SEM) from three independent experiments and statistical analyses were performed using Statistical Package for the Social Sciences version 20 (SPSS 20.0 Chicago, IL). Unless otherwise stated, statistical differences between means were determined using ANOVA test or Student t-test. *p* value < 0.05 was considered significant for all of our analyses.

## Additional Information

**How to cite this article**: Chang, W.-M. *et al*. Parathyroid Hormone-Like Hormone is a Poor Prognosis Marker of Head and Neck Cancer and Promotes Cell Growth via RUNX2 Regulation. *Sci. Rep.* **7**, 41131; doi: 10.1038/srep41131 (2017).

**Publisher's note:** Springer Nature remains neutral with regard to jurisdictional claims in published maps and institutional affiliations.

## Supplementary Material

Supplementary Figures and Tables
