# Supplementary material for: Parathyroid Hormone-Like Hormone is a Poor Prognosis Marker of Head and Neck Cancer and Promotes Cell Growth via RUNX2 Regulation
Source: Sci Rep. 2017 Jan 25;7:41131. doi: 10.1038/srep41131 (PMC5264159; doi:10.1038/srep41131)
Supplement: Supplementary Figures and Tables [file srep41131-s1.pdf]

Supplemental Information

PTHLH is a Poor Prognosis Marker of HNSCC and Promoting Cell Growth and is Regulated by RUNX2

Wei-Min Chang, Yuan-Feng Lin, Chia-Yi Su, Hsuan-Yu Peng, Yu-Chan Chang,

Jenn-Ren Hsiao, Chi-Long Chen, Jang-Yang Chang, Yi-Shing Shieh, Michael Hsiao,

Shine-Gwo-Shiah

Supplementary Figure Legends

Supplementary Figure 1. PTHLH is up-regulated in HNSCC from Oncomine™ database. The different datasets reveal that PTHLH is up-regulated in clinical HNSCC tumors comparing to normal control tissues.

Supplementary Figure 2. RUNX2 promoted PTHLH secretion.

Supplementary Figure 3. RUNX2 was not interacting with Gli2 in Ca9-22-RUNX2 Cells

Supplementary Figure 4. Roxithromycin (RXM) treatment inhibited the Cal-27 and SAS cell growth.

Supplementary Figure 5. Full length Western blot images.

Figure s1

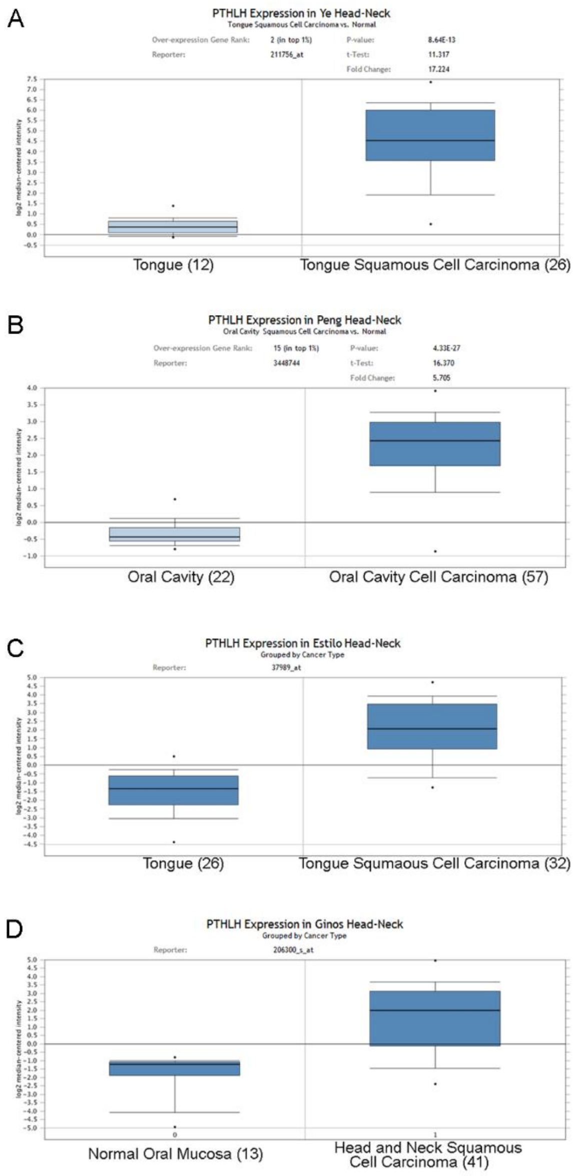

A
PTHLH Expression in Ye Head-Neck
Tongue Squamous Cell Carcinoma vs. Normal
Over-expression Gene Rank: 2 (in top 1%)
Reporter: 211756_at
P-value: 8.64E-13
t-Test: 11.317
Fold Change: 17.224
log2 median-centered intensity
Tongue (12)
Tongue Squamous Cell Carcinoma (26)
B
PTHLH Expression in Peng Head-Neck
Oral Cavity Squamous Cell Carcinoma vs. Normal
Over-expression Gene Rank: 15 (in top 1%)
Reporter: 3448744
P-value: 4.33E-27
t-Test: 16.370
Fold Change: 5.705
log2 median-centered intensity
Oral Cavity (22)
Oral Cavity Cell Carcinoma (57)
C
PTHLH Expression in Estilo Head-Neck
Grouped by Cancer Type
Reporter: 37989_at
log2 median-centered intensity
Tongue (26)
Tongue Squmaous Cell Carcinoma (32)
D
PTHLH Expression in Ginos Head-Neck
Grouped by Cancer Type
Reporter: 206300_s_at
log2 median-centered intensity
Normal Oral Mucosa (13)
Head and Neck Squamous Cell Carcinoma (41)

Figure s2

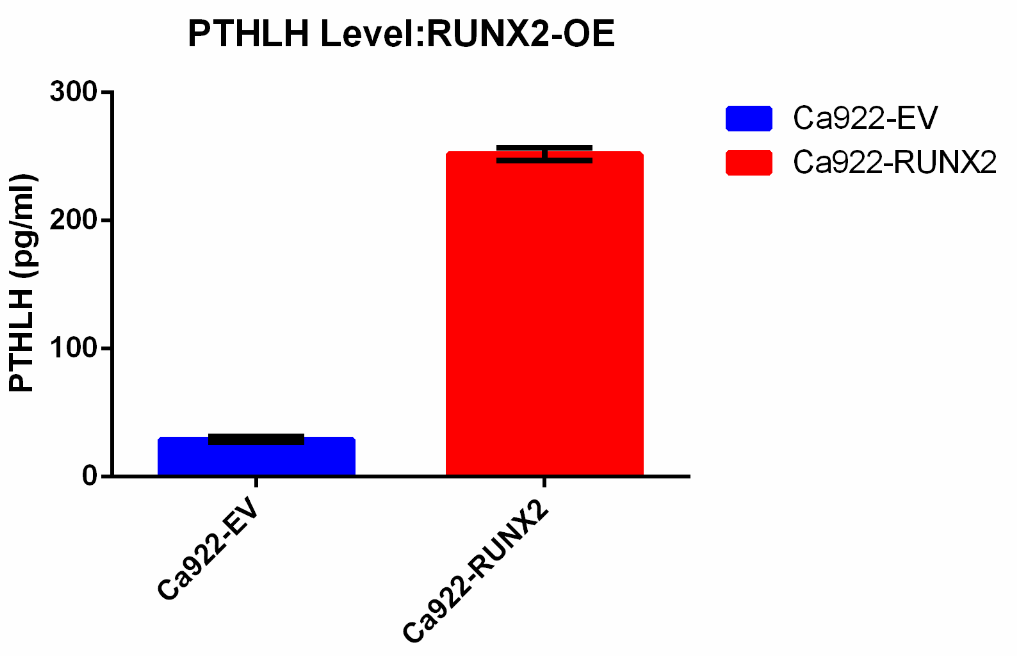
PTHLH Level:RUNX2-OE
PTHLH (pg/ml)
300
200
100
0
Ca922-EV
Ca922-RUNX2
Ca922-EV
Ca922-RUNX2

Figure s3

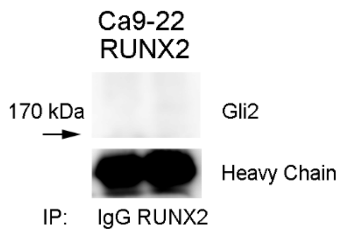

Figure s4

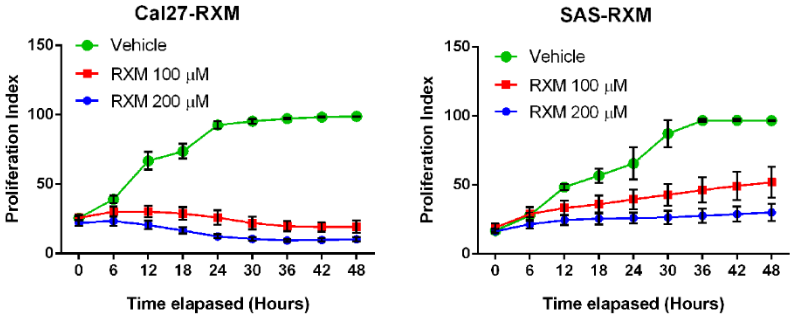

Cal27-RXM
Vehicle
RXM 100 μM
RXM 200 μM
Proliferation Index
150
100
50
0
0
6
12
18
24
30
36
42
48
Time elapsed (Hours)
SAS-RXM
Vehicle
RXM 100 μM
RXM 200 μM
Proliferation Index
150
100
50
0
0
6
12
18
24
30
36
42
48
Time elapsed (Hours)

Figure s5

A (Figure 2A)

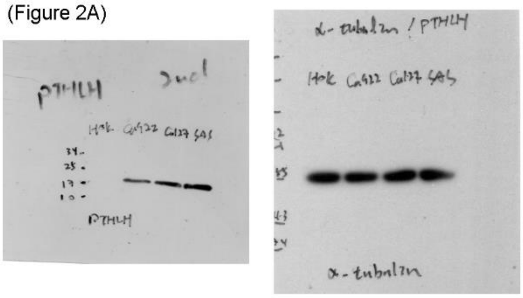

B (Figure 3D)

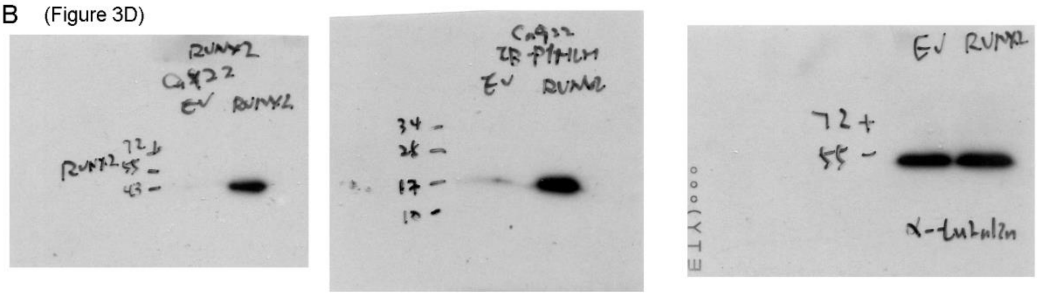

C (Figure 3F)

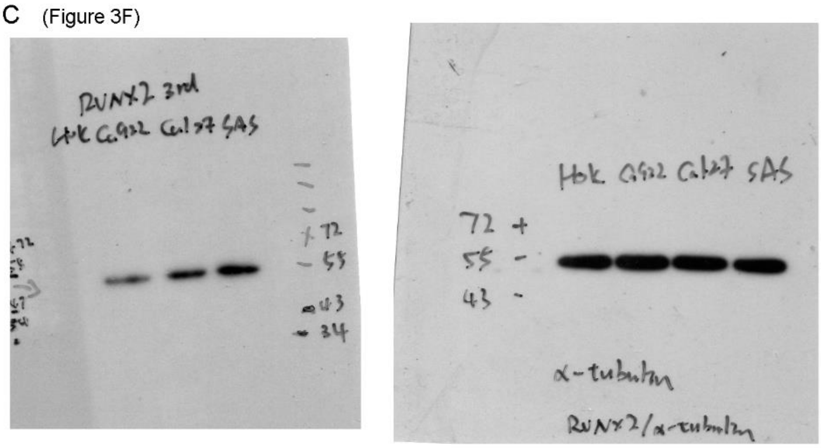

D (Figure 3K)

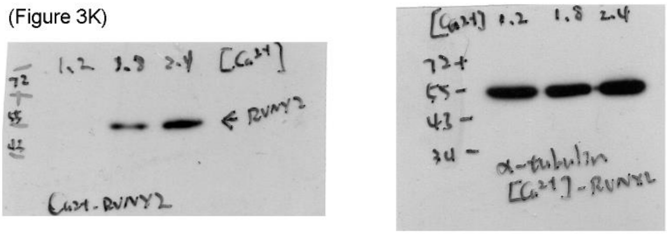

Supplementary table s1: IPA Ingenuity Canonical Pathways analysis results of PTHLH over-expression Ca9-22 cells

Supplementary table s2: Ingenuity pathway analysis revealed the difference between Cal-27 and SAS cells

Supplementary table s3: Up-regulation genes of Ca2+ pathway genes in Cal-27 cells

Supplementary table s4: Critical molecules of Cyclins and Cell Cycle Regulation Pathway that are enriched by IPA and expression pattern in GSE37991 HNSCC cohort

Supplementary table s5: Antibodies and primer list

Supplementary table s1: IPA Ingenuity Canonical Pathways analysis results of PTHLH over-expression Ca9-22 cells

Ingenuity Canonical Pathways	*-log (p-value)*	*Ratio*	*z-score*	*Molecules*
Cyclins and Cell Cycle Regulation	2.640	0.179	1.941	TP53,CCNE2,HDAC8,TFDP1,CDK6,CDKN2C,CCND1,CCNB1,CCNA2,PPM1J,PPM1L,CDKN1A,E2F5,CDC25A
Role of BRCA1 in DNA Damage Response	1.810	0.154	1.890	RAD51,FANCM,TP53,SMARCA2,CDKN1A,E2F5,SMARCE1,BRIP1,HLTF,SMARCA4,CHEK1,RFC3
Estrogen-mediated S-phase Entry	2.770	0.292	1.633	CCNA2,CCNE2,TFDP1,CDKN1A,E2F5,CCND1,CDC25A
RhoGDI Signaling	2.160	0.133	1.606	GNG4,GDI1,PAK4,GNAS,RHOC,CREBBP,GNG2,MYL5,GNB2L1,ARHGAP4,ITGA5,LIMK1,ARHGAP5,PAK1,RHOQ,ACTA2,PAK2,CD44,ARHGEF11,RHOF,ARHGEF10,GNAL,PRKCA
PI3K Signaling in B Lymphocytes	1.200	0.118	1.604	CD19,AKT2,ATF3,ATF5,RRAS,ITPR1,NFKB1,PTEN,PLCZ1,FOS,JUN,PLCG2,NFATC2,PIK3CD,PLEKHA2
Type I Diabetes Mellitus Signaling	0.650	0.100	1.342	SOCS3,HLA-DRB1,IL12B,GAD1,HLA-DMB,BID,IL1B,MAPK13,HLA-DQB1,NFKB1,HLA-E
Antioxidant Action of Vitamin C	1.400	0.131	1.265	PLCZ1,SLC2A5,PLA2G16,PLA2G4A,HMOX1,PLCG2,GPLD1,MAPK13,GSTO2,NFKB1,TXNRD1,PLD1,PLA2G12A
PPAR Signaling	1.600	0.140	1.155	FOS,IL1A,JUN,PPARD,RRAS,CREBBP,HSP90AA1,IL1B,NCOR1,PTGS2,RXRA,NFKB1,PDGFB
B Cell Activating Factor Signaling	0.720	0.125	1.000	FOS,JUN,NFATC2,MAPK13,NFKB1
Apoptosis Signaling	0.613	0.101	1.000	ACIN1,CAPN5,TP53,CAPN6,RRAS,PLCG2,BID,NFKB1,PRKCA

MIF-mediated Glucocorticoid Regulation	0.978	0.152	-1.000	PLA2G4A,LY96,PTGS2,NFKB1,PLA2G12A
Ephrin B Signaling	0.974	0.123	-1.000	GNG4,RAC2,PAK1,GNAS,ITSN1,GNB2L1,GNG2,GNAL,LIMK1
FLT3 Signaling in Hematopoietic Progenitor Cells	0.947	0.122	-1.000	PIK3R3,AKT2,RRAS,CREBBP,RPS6KA5,PIK3CD,STAT3,MAPK13,RPS6KA2
RANK Signaling in Osteoclasts	0.863	0.114	-1.000	PIK3R3,FOS,AKT2,JUN,NFATC2,PIK3CD,MAPK13,GSN,TNFRSF11A,NFKB1
Death Receptor Signaling	0.775	0.109	-1.000	ACIN1,TANK,HSPB3,TIPARP,ACTA2,ART1,BID,CFLAR,NFKB1,LIMK1
CD28 Signaling in T Helper Cells	0.710	0.102	-1.000	PIK3R3,FOS,AKT2,PAK1,JUN,HLA-DRB1,HLA-DMB,NFATC2,PIK3CD,HLA-DQB1,ITPR1,NFKB1
Calcium-induced T Lymphocyte Apoptosis	0.659	0.109	-1.000	HLA-DRB1,HLA-DMB,NR4A1,NFATC2,HLA-DQB1,ITPR1,PRKCA
FcγRIIB Signaling in B Lymphocytes	0.412	0.098	-1.000	PIK3R3,RRAS,PLCG2,PIK3CD
Non-Small Cell Lung Cancer Signaling	2.930	0.200	-1.000	PIK3R3,TP53,STK4,AKT2,TFDP1,RRAS,RARB,CDK6,PIK3CD,ITPR1,RXRA,CCND1,PRKCA
Renal Cell Carcinoma Signaling	2.570	0.183	-1.000	VEGFA,PIK3R3,FOS,PAK4,AKT2,PAK1,JUN,RRAS,CREBBP,PAK2,EGLN3,PIK3CD,PDGFB
HMGB1 Signaling	1.990	0.142	-1.000	AKT2,IL1A,RHOC,RRAS,MAPK13,NFKB1,HMGB1,PIK3R3,FOS,JUN,RHOQ,IL12B,IL1B,PIK3CD,RHOF,SERPINE1,PLAT
Glioma Invasiveness Signaling	1.540	0.158	-1.000	PIK3R3,RHOQ,RRAS,RHOC,CD44,PIK3CD,RHOF,MMP9,TIMP2
Amyloid Processing	1.400	0.157	-1.000	PRKACB,CAPN5,CAPN6,AKT2,PRKAR2B,MAPT,MAPK13,PRKAR1A
α-Adrenergic Signaling	1.470	0.138	-1.000	PRKACB,GNG4,PHKB,GNAS,PRKAR2B,RRAS,PLCG2,GNB2L1,GNG2,ITPR1,PRKCA,PRKAR1A

HIPPO signaling	1.190	0.128	-1.000	ITCH,DLG1,PPP1R14C,STK4,YAP1,RASSF6,AJUBA,PPM1J,PPM1L,CD44,CRB1
VDR/RXR Activation	1.130	0.128	-1.000	IGFBP6,COL13A1,FOXO1,PPARD,CDKN1A,NCOR1,HR,RXRA,S100G,PRKCA
mTOR Signaling	1.080	0.107	-1.000	MAPKAP1,AKT2,RHOC,RRAS,VEGFC,RPS6KA5,PLD1,PIK3R3,VEGFA,HMOX1,RHOQ,EIF4G2,EIF3B,PPM1J,PPM1L,GPLD1,PIK3CD,RPS6KA2,RHOF,PRKCA
Glioma Signaling	2.920	0.173	-1.069	TP53,AKT2,TFDP1,CAMK1D,RRAS,CDK6,CDKN2C,CCND1,PDGFB,PTEN,PIK3R3,PLCG2,CDKN1A,E2F5,IDH2,PIK3CD,PRKCA
Neuregulin Signaling	0.632	0.102	-1.134	PIK3R3,AKT2,RRAS,PLCG2,ITGA5,HSP90AA1,TMEFF2,PTEN,PRKCA
Toll-like Receptor Signaling	1.250	0.135	-1.134	SIGIRR,FOS,LY96,IL1A,JUN,TLR5,IL12B,IL1B,MAPK13,NFKB1
Role of Pattern Recognition Receptors in Recognition of Bacteria and Viruses	1.520	0.128	-1.155	OAS1,IL1A,OAS2,NFKB1,EIF2S1,IFNA14,PIK3R3,IRF7,TLR5,IL12B,PLCG2,CASP1,MAVS,IL1B,PIK3CD,PRKCA
IGF-1 Signaling	1.460	0.134	-1.155	PRKACB,PIK3R3,SOCS3,FOS,IGFBP6,AKT2,JUN,PRKAR2B,FOXO1,RRAS,PIK3CD,STAT3,PRKAR1A
RhoA Signaling	1.070	0.115	-1.155	NRP2,MYL5,ARHGAP4,MYLK,LPAR3,PLD1,LIMK1,ARHGAP5,RHPN2,ACTA2,LPAR1,PLEKHG5,SEPT10,ARHGEF11
Nitric Oxide Signaling in the Cardiovascular System	3.250	0.180	-1.213	PRKACB,AKT2,SLC7A1,VEGFC,ITPR1,PDE1C,VEGFA,PIK3R3,ADRB1,PRKG1,PRKAR2B,CAV1,HSP90AA1,PDE5A,PIK3CD,GUCY1B3,PRKAR1A,PRKCA
Tec Kinase Signaling	2.050	0.134	-1.213	GNG4,PAK4,GNAS,RHOC,GNB2L1,GNG2,ITGA5,STAT3,NFKB1,PIK3R3,FOS,P

				AK1,RHOQ,ACTA2,PLCG2,PAK2,PIK3CD,RHOF,JAK3,GNAL,PRKCA
Gα12/13 Signaling	0.386	0.086	-1.265	PIK3R3,AKT2,JUN,LPAR1,RRAS,MYL5,MEF2C,PIK3CD,NFKB1,LPAR3
NRF2-mediated Oxidative Stress Response	2.240	0.133	-1.265	DNAJB12,DNAJC9,RRAS,GSTM3,GSTA4,CREBBP,DNAJC6,DNAJC10,JUNB,DNAJA1,MAFF,TXNRD1,DNAJC11,PIK3R3,HMOX1,FOS,JUN,ACTA2,PMF1/PMF1-BGLAP,GSTM4,PIK3CD,GSTO2,ENC1,PRKCA
Cell Cycle: G1/S Checkpoint Regulation	2.050	0.172	-1.265	TP53,CCNE2,TFDP1,FOXO1,HDAC8,CDKN1A,E2F5,CDK6,CDKN2C,CCND1,CDC25A
Agrin Interactions at Neuromuscular Junction	1.430	0.145	-1.265	RAC2,PAK4,PAK1,JUN,ACTA2,RRAS,PAK2,ITGA6,ITGA5,CTTN
NF-κB Signaling	2.190	0.134	-1.279	SIGIRR,PRKACB,IL1A,AKT2,RRAS,BMP2,TGFBR3,CREBBP,FGFR2,NFKB1,TNFRSF11A,TAB3,FGFR3,TANK,PIK3R3,BRAF,TGFBR2,GHR,TLR5,PLCG2,FGFR4,IL1B,PIK3CD
Relaxin Signaling	2.830	0.156	-1.291	GNG4,PRKACB,AKT2,GNAS,GNG2,GNB2L1,PDE4A,NFKB1,PDE1C,BRAF,VEGFA,PIK3R3,FOS,PRKAR2B,JUN,PDE5A,PIK3CD,MMP9,GUCY1B3,GNAL,PRKAR1A
IL-6 Signaling	1.490	0.129	-1.291	PIK3R3,VEGFA,HSPB3,COL1A1,FOS,SOCS3,IL1A,AKT2,JUN,RRAS,IL1B,PIK3CD,MAPK13,STAT3,NFKB1
CREB Signaling in Neurons	1.410	0.117	-1.291	GNG4,PRKACB,AKT2,GNAS,GRM8,RRAS,GRID1,CREBBP,GNG2,GNB2L1,ITPR1,PIK3R3,PLCZ1,PRKAR2B,PLCG2,PIK3CD,GNAL,POLR2L,PRKAR1A,PRKCA
Oncostatin M Signaling	0.935	0.147	-1.342	RRAS,OSMR,STAT3,CHI3L1,JAK3
ErbB4 Signaling	0.505	0.100	-1.342	PIK3R3,YAP1,RRAS,PLCG2,PIK3CD,PRKCA

Actin Nucleation by ARP-WASP Complex	0.364	0.089	-1.342	RHOQ,RRAS,RHOC,ITGA5,RHOF
Cardiac Hypertrophy Signaling	1.230	0.108	-1.342	GNG4,PRKACB,GNAS,RHOC,RRAS,CREBBP,GNG2,MYL5,GNB2L1,MAPK13,ADSS,PLCZ1,TGFBR2,PIK3R3,JUN,PRKAR2B,RHOQ,ADRB1,PLCG2,MEF2C,PIK3CD,RHOF,GNAL,PRKAR1A
Wnt/β-catenin Signaling	0.813	0.101	-1.387	TP53,TCF4,AKT2,PPARD,TGFBR3,CREBBP,WNT2B,CCND1,TGFBR2,JUN,WNT7A,SOX6,PPM1J,RARB,PPM1L,CD44,FZD5
Actin Cytoskeleton Signaling	0.305	0.079	-1.387	RAC2,PAK4,RRAS,MYL5,ITGA5,MYLK,GSN,PDGFB,LIMK1,PIK3R3,PAK1,IQGAP2,CYFIP2,ACTA2,PAK2,PIK3CD,FGF5
GM-CSF Signaling	0.996	0.129	-1.414	PIK3R3,AKT2,RRAS,PIM1,GNB2L1,PIK3CD,STAT3,CCND1
Role of NANOG in Mammalian Embryonic Stem Cell Pluripotency	1.350	0.126	-1.414	TP53,AKT2,RRAS,BMP2,WNT2B,STAT3,PIK3R3,LIFR,WNT7A,GATA6,BMP7,PIK3CD,FZD5,JAK3
Integrin Signaling	3.580	0.150	-1.461	RAC2,ARHGAP26,TSPAN7,MYL5,MYLK,PTEN,BRAF,PAK1,CAV1,RHOF,CAPN5,CAPN6,PAK4,PARVA,AKT2,RHOC,RRAS,ITGA6,ITGA5,GSN,PDGFB,ARHGAP5,PIK3R3,RHOQ,ACTA2,LIMS1,PLCG2,PAK2,PIK3CD,ITGB6,CTTN
Dendritic Cell Maturation	0.696	0.096	-1.500	AKT2,IL1A,LEPR,CREBBP,CD58,MAPK13,HLA-DQB1,NFKB1,PLCZ1,PIK3R3,COL1A1,HLA-DRB1,IL12B,PLCG2,HLA-DMB,IL1B,PIK3CD
P2Y Purigenic Receptor Signaling Pathway	2.370	0.151	-1.500	GNG4,PRKACB,AKT2,GNAS,RRAS,CREBBP,GNG2,GNB2L1,NFKB1,PLCZ1,PIK3R3,FOS,PRKAR2B,JUN,PLCG2,PIK3CD,PRKAR1A,PRKCA

Rac Signaling	2.240	0.154	-1.500	PAK4,RRAS,ITGA5,NFKB1,PLD1,LIMK1,PIK3R3,MCF2L,PAK1,IQGAP2,CYFIP2,JUN,PAK2,CD44,PIK3CD,ELK4
Synaptic Long Term Depression	1.100	0.113	-1.500	PLA2G16,GNAS,GRM8,RRAS,GRID1,ITPR1,PLCZ1,PLA2G4A,PRKG1,PPM1J,PLCG2,PPM1L,GUCY1B3,GNAL,PLA2G12A,PRKCA
Phospholipase C Signaling	1.180	0.105	-1.500	GNG4,GNAS,HDAC8,RRAS,RHOC,CREBBP,GNG2,GNB2L1,MYL5,ITGA5,ITPR1,NFKB1,PLD1,PLA2G4A,HMOX1,RHOQ,PLCG2,GPLD1,NFATC2,MEF2C,ARHGEF11,RHOF,ARHGEF10,PRKCA,PLA2G12A
14-3-3-mediated Signaling	1.190	0.120	-1.508	TUBA1B,AKT2,YAP1,RRAS,PLCZ1,PIK3R3,FOS,JUN,FOXO1,PLCG2,MAPT,TUBA1C,PIK3CD,PRKCA
CDK5 Signaling	1.110	0.121	-1.508	PRKACB,PPP1R14C,GNAS,PRKAR2B,RRAS,PPM1J,MAPT,PPM1L,ITGA6,MAPK13,GNAL,PRKAR1A
ERK/MAPK Signaling	3.300	0.150	-1.512	PRKACB,HSPB3,RAC2,DUSP6,BRAF,PAK1,ELF3,ETS2,PPM1J,PPM1L,MKNK1,PRKCA,PLA2G12A,PPP1R14C,PAK4,RRAS,CREBBP,ITGA5,RPS6KA5,STAT3,PIK3R3,PLA2G4A,FOS,PRKAR2B,PLCG2,PAK2,PIK3CD,PRKAR1A
Role of NFAT in Cardiac Hypertrophy	1.460	0.117	-1.606	GNG4,PRKACB,AKT2,GNAS,HDAC8,CAMK1D,RRAS,GNG2,GNB2L1,ITPR1,MAPK13,PLCZ1,PIK3R3,TGFBR2,PRKAR2B,PLCG2,MEF2C,PIK3CD,RCAN2,PRKAR1A,PRKCA
IL-2 Signaling	0.960	0.132	-1.633	PIK3R3,FOS,AKT2,JUN,RRAS,PIK3CD,JAK3
LPS-stimulated MAPK Signaling	0.974	0.123	-1.667	PIK3R3,FOS,PAK1,JUN,RRAS,PIK3CD,MAPK13,NFKB1,PRKCA
TREM1 Signaling	0.920	0.120	-1.667	SIGIRR,AKT2,TLR5,PLCG2,CASP1,ITGA5,IL1B,STAT3,NFKB1

Aldosterone Signaling in Epithelial Cells	2.200	0.138	-1.667	HSPB3,CRYAB,DNAJB12,DNAJC9,HSPH1,SLC12A2,HSPA9,DNAJC6,DNAJC10,ITPR1,DNAJA1,DNAJC11,SCNN1A,PLCZ1,PIK3R3,HSPA8,PLCG2,ASIC1,HSP90AA1,PIK3CD,PRKCA
EGF Signaling	1.590	0.161	-1.667	PIK3R3,FOS,AKT2,JUN,PIK3CD,STAT3,MAPK13,ITPR1,PRKCA
VEGF Signaling	1.310	0.130	-1.667	VEGFA,PIK3R3,AKT2,FOXO1,ACTA2,RRAS,PLCG2,VEGFC,PIK3CD,EIF2S1,ELAVL1,PRKCA
Pancreatic Adenocarcinoma Signaling	4.820	0.208	-1.698	TP53,AKT2,TFDP1,VEGFC,STAT3,NFKB1,BIRC5,CCND1,PLD1,RAD51,TGFBR2,PIK3R3,VEGFA,HMOX1,CYP2E1,CDKN1A,GPLD1,E2F5,PIK3CD,PTGS2,JAK3,MMP9
CXCR4 Signaling	2.200	0.138	-1.698	GNG4,PAK4,AKT2,GNAS,RHOC,RRAS,MYL5,GNB2L1,GNG2,ITPR1,PIK3R3,FOS,PAK1,JUN,RHOQ,PAK2,ARHGEF11,PIK3CD,RHOF,GNAL,PRKCA
Signaling by Rho Family GTPases	1.430	0.111	-1.706	GNG4,PAK4,GNAS,RHOC,GNG2,GNB2L1,MYL5,ITGA5,MYLK,NFKB1,PLD1,LIMK1,PIK3R3,STMN1,FOS,PAK1,RHOQ,JUN,ACTA2,PAK2,SEPT10,PIK3CD,ARHGEF11,RHOF,ARHGEF10,GNAL
p70S6K Signaling	0.693	0.101	-1.732	PLCZ1,PIK3R3,CD19,AKT2,RRAS,PPM1J,PLCG2,MAPT,PPM1L,PIK3CD,PLD1,PRKCA
UVA-Induced MAPK Signaling	2.560	0.170	-1.732	TP53,RRAS,ART1,RPS6KA5,MAPK13,PLCZ1,PIK3R3,FOS,JUN,SMPD4,TIPARP,PLCG2,PIK3CD,RPS6KA2,PRKCA
Regulation of Actin-based Motility by Rho	1.670	0.143	-1.732	RAC2,PAK4,PAK1,RHOQ,ACTA2,RHOC,MYL5,PAK2,ITGA5,MYLK,GSN,RHOF,LIMK1
PAK Signaling	1.410	0.135	-1.732	PIK3R3,PAK4,PAK1,RRAS,MYL5,PAK2,ITGA5,MYLK,PIK3CD,EPHA3,PDGFB,LIMK1
IL-8 Signaling	3.410	0.152	-1.800	RAC2,GNB2L1,NFKB1,CCND1,LIMK1,VEGFA,BRAF,HMOX1,JUN,GPLD1,RHOF

				,PRKCA,GNG4,AKT2,GNAS,ANGPT1,RHOC,RRAS,GNG2,VEGFC,PLD1,PIK3R3,FOS,RHOQ,PAK2,PIK3CD,PTGS2,MMP9
eNOS Signaling	3.290	0.162	-1.886	PRKACB,AKT2,GNAS,HSPA9,SLC7A1,VEGFC,ITPR1,LPAR3,HSPA8,PIK3R3,VEGFA,CCNA2,PRKG1,PRKAR2B,CNGB1,LPAR1,PLCG2,CAV1,HSP90AA1,PIK3CD,GUCY1B3,PRKAR1A,PRKCA
Glioblastoma Multiforme Signaling	2.410	0.144	-1.886	TP53,AKT2,RHOC,RRAS,WNT2B,CDK6,ITPR1,CCND1,PDGFB,PTEN,PIK3R3,PLCZ1,WNT7A,RHOQ,FOXO1,PLCG2,CDKN1A,E2F5,FZD5,PIK3CD,RHOF
CNTF Signaling	0.994	0.135	-1.890	PIK3R3,LIFR,RRAS,RPS6KA5,PIK3CD,STAT3,RPS6KA2
Melatonin Signaling	0.776	0.114	-1.890	PLCZ1,PRKACB,BRAF,PRKAR2B,RORA,PLCG2,PRKCA,PRKAR1A
ERK5 Signaling	0.682	0.111	-1.890	FOS,RRAS,CREBBP,MEF2C,RPS6KA5,RPS6KA2,ELK4
Macropinocytosis Signaling	1.120	0.132	-1.890	PIK3R3,PAK1,RRAS,PLCG2,ITGA5,PIK3CD,ITGB6,PDGFB,PRKCA
Fcγ Receptor-mediated Phagocytosis in Macrophages and Monocytes	0.754	0.108	-1.897	PIK3R3,HMOX1,RAC2,AKT2,PAK1,ACTA2,GPLD1,PLD1,PTEN,PRKCA
Fc Epsilon RI Signaling	0.499	0.093	-1.897	PIK3R3,PLA2G4A,RAC2,AKT2,RRAS,PLCG2,PIK3CD,MAPK13,PRKCA,PLA2G12A
Prolactin Signaling	2.030	0.164	-1.897	PIK3R3,SOCS3,FOS,JUN,RRAS,PLCG2,CREBBP,NMI,PIK3CD,STAT3,TCF7,PRKCA
Growth Hormone Signaling	1.430	0.145	-1.897	PIK3R3,SOCS3,FOS,GHR,PLCG2,RPS6KA5,PIK3CD,STAT3,RPS6KA2,PRKCA
HGF Signaling	2.200	0.152	-1.941	AKT2,RRAS,ITGA5,STAT3,CCND1,PIK3R3,FOS,PAK1,ELF3,JUN,ETS2,PLCG2,CDKN1A,PIK3CD,PTGS2,PRKCA

G Beta Gamma Signaling	2.150	0.159	-1.941	PRKACB,GNG4,AKT2,GNAS,RRAS,GNB2L1,GNG2,PAK1,PRKAR2B,PLCG2,CAV1,GNAL,PRKAR1A,PRKCA
Neuropathic Pain Signaling In Dorsal Horn Neurons	1.370	0.130	-1.941	PRKACB,CAMK1D,GRM8,ITPR1,PIK3R3,PLCZ1,FOS,PRKAR2B,GPR37,PLCG2,PIK3CD,PRKCA,PRKAR1A
Sperm Motility	1.950	0.140	-2.000	PLA2G16,PRKACB,GNAS,SLC12A2,PDE4A,ITPR1,PDE1C,PLCZ1,PLA2G4A,CNGB1,PRKG1,PRKAR2B,PLCG2,GUCY1B3,PRKAR1A,PRKCA,PLA2G12A
PCP pathway	0.965	0.127	-2.121	JUN,WNT7A,WNT2B,CTHRC1,FZD5,JUNB,PRICKLE1,LGR4
Chemokine Signaling	0.752	0.113	-2.121	FOS,JUN,CAMK1D,RRAS,PLCG2,MAPK13,PRKCA,LIMK1
MIF Regulation of Innate Immunity	1.940	0.195	-2.121	TP53,PLA2G4A,FOS,LY96,JUN,PTGS2,NFKB1,PLA2G12A
Thrombopoietin Signaling	1.240	0.145	-2.121	PIK3R3,FOS,JUN,RRAS,PLCG2,PIK3CD,STAT3,PRKCA
Thrombin Signaling	1.960	0.126	-2.236	GNG4,AKT2,GNAS,CAMK1D,RHOC,RRAS,GNG2,MYL5,GNB2L1,MYLK,ITPR1,MAPK13,NFKB1,PLCZ1,PIK3R3,RHOQ,PLCG2,GATA6,ARHGEF11,PIK3CD,RHOF,ARHGEF10,GNAL,PRKCA
Huntington's Disease Signaling	1.320	0.109	-2.236	GNB2L1,NAPG,JUN,CASP1,NCOR1,POLR2L,PRKCA,TP53,GNG4,CAPN5,CAPN6,AKT2,HDAC8,PSMF1,SH3GL3,HSPA9,IFT57,GNG2,CREBBP,ITPR1,PSME3,PIK3R3,HSPA8,HAP1,PIK3CD
PDGF Signaling	1.850	0.156	-2.309	PIK3R3,FOS,JUN,RRAS,PLCG2,CAV1,SPHK1,PIK3CD,STAT3,JAK3,PDGFB,PRKCA
Regulation of Cellular Mechanics by Calpain Protease	1.160	0.140	-2.449	CAPN5,CCNA2,CAPN6,CNGB1,RRAS,CDK6,ITGA5,CCND1

Colorectal Cancer Metastasis Signaling	3.880	0.148	-2.475	PRKACB,TCF4,GNB2L1,NFKB1,CCND1,VEGFA,BRAF,TGFBR2,JUN,WNT7A,RHOF,PTGER4,TP53,GNG4,AKT2,GNAS,RHOC,RRAS,ADRBK2,GNG2,WNT2B,VEGFC,STAT3,BIRC5,PIK3R3,FOS,PRKAR2B,RHOQ,TLR5,FZD5,PIK3CD,PTGS2,JAK3,MMP9,PRKAR1A
Endothelin-1 Signaling	2.490	0.140	-2.558	PLA2G16,GNAS,RRAS,PTGS1,ITPR1,MAPK13,PLD1,BRAF,PIK3R3,PLCZ1,PLA2G4A,FOS,HMOX1,JUN,EDN1,PLCG2,GPLD1,CASP1,PIK3CD,PTGS2,GUCY1B3,GNAL,PRKCA,PLA2G12A
VEGF Family Ligand-Receptor Interactions	1.890	0.158	-2.714	VEGFA,PIK3R3,PLA2G4A,FOS,AKT2,NRP2,RRAS,PLCG2,VEGFC,PIK3CD,PRKCA,PLA2G12A
Acute Phase Response Signaling	1.710	0.124	-2.828	SOCS3,TCF4,IL1A,AKT2,RRAS,AHSG,AMBP,SERPINA3,STAT3,MAPK13,NFKB1,PIK3R3,HMOX1,FOS,JUN,IL1B,SERPINA1,OSMR,PIK3CD,SERPINE1,C2
Cholecystokinin/ Gastrin-mediated Signaling	1.060	0.119	-2.887	FOS,IL1A,JUN,RHOQ,RRAS,RHOC,IL1B,MEF2C,PTGS2,ITPR1,RHOF,PRKCA

Supplementary table s2: Ingenuity pathway analysis revealed the difference between Cal-27 and SAS cells

Cal-27 chemical - endogenous mammalian

Upstream Regulator	Predicted Activation State	Activation z-score	p-value of overlap
tretinoin	Activated	3.884	6.35E-09
Ca2+	Activated	3.597	0.000203
hydrogen peroxide	Activated	3.049	0.00225
arachidonic acid	Activated	2.919	0.0253
D-galactosamine	Activated	2.63	0.00273
sphingosine-1-phosphate	Activated	2.411	0.201
palmitic acid	Activated	2.407	1
nitric oxide	Activated	2.223	0.00798
cholesterol	Activated	2.212	0.0681
ceramide	Activated	2.2	0.00133
melatonin	Activated	2.2	0.438
platelet activating factor	Activated	2.183	0.0958
linoleic acid	Activated	2.169	0.197
quinolinic acid	Activated	2.164	0.0162
formaldehyde	Activated	2.138	0.009
leukotriene D4	Activated	2.135	0.00393
aldosterone	Activated	2.042	0.379

SAS chemical - endogenous mammalian

Upstream Regulator	Predicted Activation State	Activation z-score	p-value of overlap
phosphate	Activated	2	0.22
dinoprost		1.982	0.0505
testosterone		1.44	0.0307
beta-estradiol		0.867	0.0011
dihydrotestosterone		0.654	0.0264
thyroid hormone		0.294	0.00011
tretinoin		-0.222	0.000188
niacinamide		-1	0.0305
dopamine		-1.987	0.289

Supplementary table s3: Up-regulation genes of Ca^{2+} pathway genes in Cal-27 cells

AHR,APEX1,ATF3,CDK2,CDK4,CREM,DUOX1,E2F3,EGFR,FABP4,FLG2,FOS,ITGA6,
JUN,JUNB,KLF4,KRT1,KRT10,MGP,PTH,S100A7,S100A8,S100A9,SBSN,SPRR2A,TGM1

Supplementary table s4: Critical molecules of Cyclins and Cell Cycle Regulation Pathway that are enriched by IPA and expression pattern in GSE37991 HNSCC cohort

Symbol	PTHLH Array Log2 Value	Expected Regulation	GSE37991 T/N Ratio	Consistency
CCNA2	1.873	Up	1.50	V
CCND1	1.582	Up	0.87	
CCNE2	1.818	Up	2.13	V
CDC25A	1.616	Up	1.66	V
CDKN1A	-1.541	Down	2.28	
E2F5	-1.617	Up	1.03	
TFDP1	1.916		1.17	

Supplementary table s5: Antibodies and primer list

Antibody

Symbol	Brand	Catalog Number
RUNX2	Santa Cruz	sc-101145
RUNX2 (IP)	Santa Cruz	sc-10758X
GLI2	Cell Signaling	#2585
PTHLH	Genetex	GTX108405
a-Tubulin	Sigma	T9026

Primer

Primer Name	Sequence
GAPDH-F	GAAGGTGAAGGTCGGAGT
GAPDH-R	GAAGATGGTGATGGGATTTC
RUNX2-F	CCGCCTCAGTGATTTAGGGC
RUNX2-R	GGGTCTGTAATCTGACTCTGTCC
PTHLH-F	AAGGTGGAGACGTACAAAGAGC
PTHLH-R	CAGAGCGAGTTCGCCGTTT
Primer for UPL assay	Sequence
GAPDH-F-UPL#60	AGCCACATCGCTCAGACAC
GAPDH-R-UPL#60	GCCCAATACGACCAAATCC
RUNX2-F-UPL#4	GTGCCTAGGCGCATTTCA
RUNX2-R-UPL#4	GCTCTTCTTACTGAGAGTGGAAGG
Primer for ChIP assay	Sequence
RUNX2-ChIPQ-PTHLH-800-F	AAATTCACAGGCCAACAGCC
RUNX2-ChIPQ-PTHLH-800-R	CGAGGAGGAGGCCAAGGA
RUNX2-ChIPQ-PTHLH-500-F	TCCGGGGTCGGCAGC
RUNX2-ChIPQ-PTHLH-500-R	AGCGAGTGGAGGGGAGC
